# Reducing overdose after release from incarceration (ROAR): study protocol for an intervention to reduce risk of fatal and non-fatal opioid overdose among women after release from prison

**DOI:** 10.1186/s40352-020-00113-7

**Published:** 2020-07-10

**Authors:** Elizabeth Needham Waddell, Robin Baker, Daniel M. Hartung, Christi J. Hildebran, Thuan Nguyen, Deza’Rae M. Collins, Jessica E. Larsen, Erin Stack, the ROAR Protocol Development Team, Tina Bialas, Sarann Bielavitz, Jessica Gregg, P. Todd Korthuis, Lynn Kunkel, Joshua D. Lee, Gillian Leichtling, Dawnell L. Meyer, Ceilidh Nichols, Katharina Wiest

**Affiliations:** 1 Oregon Health & Science University-Portland State University School of Public Health, 3181 SW Sam Jackson Park Rd, CB669, Portland, OR 97239 USA; 2 College of Pharmacy – Oregon State University/Oregon Health & Science University, Robertson Collaborative Life Sciences Building (RLSB), 2730 SW Moody Ave., CL5CP, Portland, OR 97201-5042 USA; 3 Comagine Health, 650 NE Holladay St., Suite 1700, Portland, OR 97232 USA

**Keywords:** opioid-related disorders, drug overdose, Vivitrol, justice-involved women, recovery mentors, peer navigators, MAT, MOUD, correctional health, substance use disorder treatment

## Abstract

**Background:**

Drug overdose is the leading cause of death after release from prison, and this risk is significantly higher among women compared to men. Within the first 2 weeks after release, the risk of death from drug overdose is 12.7 times higher than the general population, with risk of death further elevated among females. Although female inmates have higher rates of opioid use disorder and post-release overdose fatality, justice-involved women are under-represented in studies of medications for opioid use disorder. The Reducing Overdose After Release from Incarceration (ROAR) pilot intervention and evaluation (recruitment June 2019 through December 2020) aims to reduce opioid overdose among women released to the community following incarceration in state prison. The evaluation further assesses induction, acceptance and effectiveness of extended release naltrexone in a female post-prison population.

**Methods/design:**

In the week prior to their release, female adults in custody with moderate to severe opioid use disorder start treatment with extended release naltrexone, an injectable opioid antagonist that blocks the effects of opioids for up to 1 month. All ROAR participants receive training to use naloxone rescue kits and are provided nasal naloxone at release. Ongoing support from a certified recovery mentor to facilitate sustained engagement with treatment for substance use disorders begins in the month prior to release from prison and continues for 6 months in community. We evaluate the association between ROAR participation and the primary outcome of opioid overdose.

Using administrative data provided by the Oregon Department of Corrections and the Oregon Health Authority, we compare the odds of overdose among ROAR participants versus a comparison group of females released from prison during the study period. Evaluation activities in community includes survey and qualitative interviews for 6 months post release, as well as a review of clinic records to assess retention on medication among the pilot cohort (*N* = 100).

**Discussion:**

ROAR is a collaboration between Oregon’s public health, criminal justice, and medical communities. The ROAR intervention and evaluation provide critical information on improving interventions to prevent opioid overdose and improve retention on treatment in community in an overlooked, high-risk population: incarcerated women re-entering the community.

**Trial registration:**

Clinical Trials.gov TRN: NCT03902821.

## Background

### Opioid overdose risk among justice-involved women

Risk for drug-related death is significantly elevated in the 4 weeks following release from prison (Merrall et al., 2010). Among adults released from Washington State prisons between 1999 and 2003, the all-cause risk of death in the 2 weeks post release was 12.7 times greater than for other residents, and drug overdose was the leading cause of death after release (Binswanger et al., 2016). Drug overdose death after release from incarceration has been linked to multiple individual and social determinants. Individual risk factors for overdose include a decrease in drug tolerance while incarcerated due to no drug access or access to low purity drugs (Merrall et al., 2010). Risks of overdose are further elevated when heroin is used with other substances (e.g., alcohol, benzodiazepines, and other depressants) (Coffin et al., 2007), there is presence of comorbid medical disorders (e.g., HIV and mental health disorders) (Coffin et al., 2007), and there is a history of prior overdose (Coffin et al., 2007) or injection of drugs (Merrall et al., 2010). Formerly incarcerated individuals, moreover, may be exposed to socio-cultural norms or behaviors that encourage drug use (i.e., drug use to celebrate release). Little to no access to treatment facilities, a lack of social networks, and homelessness also increase overdose risk (Merrall et al., 2010).

The burden and adverse consequences of substance use disorders (SUD) are substantially higher in the female inmate population compared to males. In 2016, 25% of incarcerated women were convicted of a drug offense compared to 15% of incarcerated men (Carson, Mueller, & Kaeble, 2018). Compared to men, incarcerated women are significantly more likely to have severe substance abuse histories, co-occurring mental disorders and physical health problems, and histories of trauma and abuse (Rettinger & Andrews, 2010; Van Voorhis, 2012; Van Voorhis, Wright, Salisbury, & Bauman, 2010). In the United States, women in the criminal justice system are more likely than men to abuse and be addicted to heroin and opioids (Evans, 2015). According to prison intake data collected in the past two years, roughly half of women entering prison in Oregon used heroin, street methadone, or other opioids in the 12 months prior to incarceration. Incarcerated women who have a history of opioid-addiction are more likely than men are to inject opioids (Evans, 2015). Moreover, women in prison have higher HIV rates compared to men (Galea & Vlahov, 2002). Incarcerated women are more likely to use opioids daily and have increased risk for an opioid overdose post-release (Evans, 2015). Finally, women are more susceptible to overdose than men for biological reasons , including lower tolerance leading to both physical dependence and overdose with small quantities of substance (Springer, Biondi, Frank, & El-Bassel, 2020). Although each of these risks make women more susceptible to drug overdose post-incarceration (Binswanger et al., 2007), unique treatment needs post-release and gender specific treatments for OUD and recovery support for incarcerated women have not been well studied (Evans, 2015).

A common barrier to initiating treatment, as reported in a population of justice-involved veterans, includes limited availability of pharmacotherapy treatment options (Finlay et al., 2016). Treatment retention is further impacted by housing insecurity, lack of financial resources, exposure to actively using friends and family, and lack of knowledge; these barriers emphasize the need for post-incarceration treatment plans that clearly specify medications for opioid use disorders (MOUD) (Velasquez et al., 2019). Another barrier is providers who are not supportive of pharmacotherapy (Harris et al., 2012). Clinical staff who are supportive of pharmacotherapy play a crucial role in treatment retention by facilitating a welcoming patient environment. Gender specific barriers include the stigmatization of women with a diagnosed opioid use disorder (OUD) due to their role in society as caretakers and possibly having to choose between drug treatment and continuing to care for children (van Olphen, J., Eliason, M., Freudenberg, N., & Barnes, M., 2009).

### Extended-release naltrexone (XR-NTX) for prevention of overdose

There are presently three medications for treatment of OUD that are approved by the Food and Drug Administration: methadone (opioid agonist dispensed only in certified opioid treatment programs), buprenorphine (partial opioid agonist dispensed by providers with special training for a limited number of patients) and naltrexone (a full opioid antagonist dispensed by any licensed provider) (Jarvis et al., 2018). The present study evaluates a voluntary pilot intervention offering induction on XR-NTX to women with a moderate to severe OUD. Induction takes place in the prison health services in the week prior to release. The injectable formulation of XR-NTX was selected for the pilot intervention because its administration is permitted by nursing staff without special certifications and because the target population is assumed to be opioid free at the time of release. Results will inform the state’s implementation of future interventions to treat OUD, including both agonist and antagonist medications.

Administering XR-NTX prior to release from incarceration is known to improve treatment retention rates compared to administering XR-NTX post-release, but additional studies are needed to determine the efficacy of naltrexone use in combination with community-based services (Lincoln, Johnson, McCarthy, & Alexander, 2018). The most recent systematic review of published literature on the induction, acceptability and decrease in opioid use associated with XR-NTX found that XR-NTX showed show similar efficacy to buprenorphine when randomization occurred post detoxification (Jarvis et al., 2018). Failure to start XR-NTX, especially among populations requiring opioid detoxification prior to induction is found to be a significant barrier to its effectiveness in community treatment settings. Successful induction and retention may be more likely when offered in corrections settings prior to release (Friedmann, Wilson, Hoskinson Jr., Poshkus, & Clarke, 2018; Lincoln et al., 2018) and among patients who have already completed detoxification (Tanum et al., 2017).

Jarvis et al’s systematic review (2018) did not include any studies with sufficient power to determine efficacy of XR-NTX for prevention of overdose compared to other forms of treatment. The utilization of XR-NTX for the prevention of overdose is often an overarching study aim in OUD interventions, but standards for reporting actual cases of overdose are not always consistent (Jarvis et al. 2018). Meta-analysis of 22 studies administering XR-NTX reported opioid use as an outcome while 15 of those studies also reported overdose as an outcome (Jarvis et al., 2018). Of those, 60% did not report any case of overdose among participants. The remaining six studies reported both fatal and non-fatal overdose at rates ranging between 3.5% (2 of 57), 4.0% (1 of 25) and 5.3% (15 of 283) for non-fatal overdose and as low as 0.7% ( 1 of 150), 0.7% ( 2 of 283), 1.2% ( 2 of 171) and high as 4.5% ( 3 of 67) for fatal overdose. While XR-NTX and other MOUD delay return to opioid use in justice-involved populations and may reduce risk of overdose, the largest trial of XR-NTX among criminal justice offenders (*N* = 308) included just 15% female participants (Lee et al., 2016). In fact very few women have participated in MOUD treatment engagement and retention studies (Springer et al., 2020). Reports of women’s participation in XR-NTX treatment and retention are generally lacking, however exposure to oral methadone or buprenorphine while incarcerated has resulted in a 75% decrease in all-cause mortality and 85% reduction in drug related poisoning 4 weeks after release in England's adult women's prison population (Marsden et al., 2017).

As Springer et al. (2020) have recommended, we need new research to understand how women access and maintain SUD treatment including MOUD. We need more research in order to guide the field toward development of evidence-based interventions to reduce known barriers to treatment among women, including but not limited to violence and trauma, needs for child care and transportation, and fear of laws that punish women who use drugs (Springer et al., 2020).

Broadening scientific understanding about the feasibility and acceptability of XR-NTX to treat OUD among justice-involved women holds great public health significance. The ROAR pilot’s setting in an all-female prison will provide new information on barriers and facilitators of induction pre-release, as well as feasibility of retention on treatment in four Oregon counties in which local Medicaid covers treatment with all three forms of MOUD. Agency partners employ Certified Recovery Mentors (CRM) to facilitate transition to treatment post release. CRMservices are also billable to Medicaid in Oregon.

### Peer-supported recovery for justice-involved women

Effective re-entry programming that includes interpersonal interactions with community social services is key to reducing return to incarceration (Kendall, Redshaw, Ward, Wayland, & Sullivan, 2018) and risk of overdose for individuals with OUD. Through the ROAR pilot, the Oregon Department of Corrections offers XR-NTX to female inmates with moderate to severe OUD prior to release from incarceration. ROAR adds peer-support recovery services provided by specifically trained female CRMs with lived experience to improve access to continued MOUD. Peer Based Recovery Support Services (White, 2009) have a long and valuable history with (SUD) treatment and in assisting individuals seeking recovery. Peer recovery mentors began in the late 1930’s and 40’s within mutual-aid (12 step) groups, specifically serving as “sponsors” to individuals seeking recovery from alcoholism. Mental health peer mentors made gains in the early 1980’s with the mental health consumer movement, where individuals with lived experience with mental illness, who achieved some degree of recovery, used their personal experiences to help others. Individuals’ recovery from alcohol and drugs and consumers of mental health services have a long history serving as peer mentors assisting another person’s recovery, by instilling hope, modeling recovery behavior and supporting individuals in their daily life without stigma. Peers have been defined as “offering and receiving help, based on shared understanding, respect, and mutual empowerment between people in similar situations” (Mead, Hildton, & Curtis, 2001).

ROAR’s peer model differs from most commonly cited peer supports in that it includes formal, structured visits that are and intensively goal-oriented (Bagnall et al., 2015). The ROAR CRM specific model includes assisting woman in establishing recovery goals and identifying barriers to achieving those goals early on. More specifically, the ROAR  CRM aids with ongoing assessment of safe and stable housing along with the continued goals of supporting pro-social behaviors and addressing criminogenic risk factors such as criminal thinking and antisocial attitudes and beliefs (Heidemann, Cederbaum, & Martinez, 2014). ROAR CRMs work within the principled framework of recovery-oriented, person-centered, relationship-focused and trauma-informed care and encourage strong social networks. They assist with irecovery efforts as well as more general activities of daily living.

Little is known about the effectiveness of professionally certified peer supports for formerly incarcerated individuals, particularly for women with OUD, who are transitioning into the community (Green et al., 2018; Lee et al., 2016; Lincoln et al., 2018). Reviews of strategies to support justice-involved women rated interventions that include forensic peer support as promising (Heiss, Somers, & Larson, 2016) and having a medium-to-high impact (Dorn et al., 2018). These ratings are supported by early studies which have observed that justice-involved people who work with forensic peers are very satisfied with peers (Baron, 2011), have increased predicted life satisfaction (Heidemann et al., 2014), decreased rates of emergency department utilization (Wang et al., 2012), and decreased substance misuse and offending rates (Mowen & Boman IV, 2018). In addition, forensic peers providing services report positive self-reflection as part of being able to “give back” (Baron, 2011) and increased self-esteem, prosocial behavior, and social connectedness (Heidemann, Cederbaum, Martinez, & LeBel, 2016). Forensic peer interventions may be especially important for justice-involved women compared to men, as women demonstrate an increased need for social support post-incarceration (Pettus-Davis, Veeh, Davis, & Tripodi, 2018; Valera, Chang, Hernández, & Cooper, 2015).

### ROAR study objectives

Facilitating connections between newly released justice-involved populations and community-based services or programs improves SUD treatment adherence (Heiss et al., 2016) and reduces risk of opioid overdose (Marlowe, Wakeman, Rich, & Baston, 2016). Evidence-based strategies known to be effective include MOUD and relapse prevention (Heiss et al., 2016). Promising strategies include case management, where support services are coordinated, and mutual aid or 12-step group programs to support individuals in recovery.

ROAR draws on aspects of MOUD, peer support from CRMs, and mutual aid to reduce fatal and non-fatal overdose among formerly incarcerated women upon release (Heiss et al., 2016). The project is funded by the Centers for Disease Control and Prevention as a pilot program to reduce fatal and non-fatal opioid overdose and has two primary aims: 1) pilot the intervention to reduce risk of overdose among adult women released from prison and 2) evaluate the effect of the pilot project on opioid overdose among adult women released from prison.

## Methods

### Study design

**Fig. 1:**
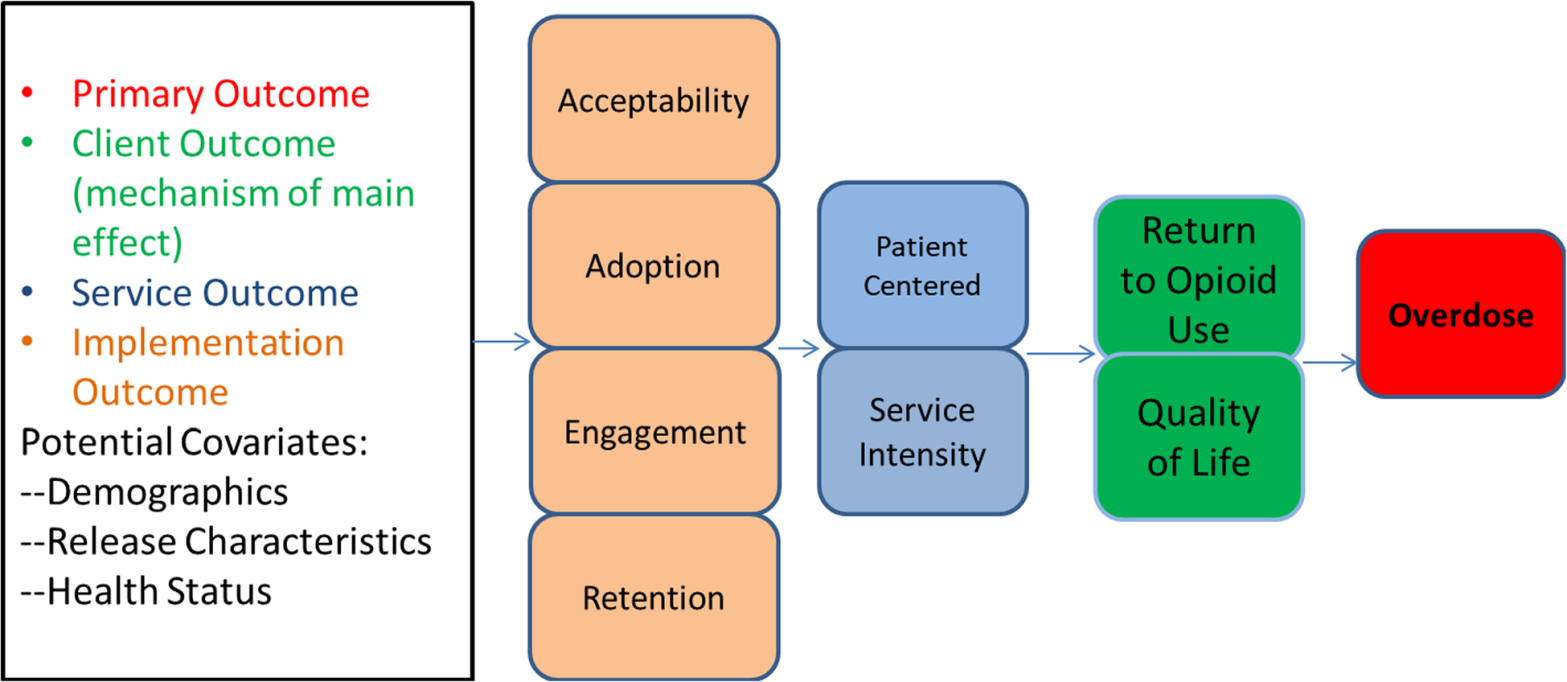
Conceptual model for ROAR pilot evaluation

ROAR aims to reduce overdose and to assess feasibility, acceptability, and satisfaction with a range of community treatment options available to women after they are released to community in one of four pilot counties comprising two urban centers in Oregon. See Fig. 1: Conceptual Model for ROAR Pilot Evaluation.

Aim 1 collects participant survey data, completes qualitative interview data, and abstracts medical charts to assess: 1) Implementation Outcomes, 2) Service Outcomes, and 3) Client Outcomes. Implementation outcomes examine acceptability, adoption, engagement and retention in the ROAR pilot. Aim 2 evaluates the effects of the ROAR program on our primary outcome of any opioid overdose. This is a binary outcome indicating a fatal or non-fatal opioid-related overdose event in the 6 months following release from incarceration.

#### Aim 1

Pilot the ROAR intervention to reduce risk of overdose among adult women released from prison. ROAR is offered to all eligible female inmates being released to one of Oregon’s four most populated counties over the 18-month recruitment period. The pilot (*n* = 100) includes a process evaluation using quantitative and qualitative measures grounded in the Consolidated Framework for Implementation Research (Keith, Crosson, O'Malley, Cromp, & Taylor, 2017). Baseline participant surveys assess history of overdose, lifetime mental health status and current depression using the Patient Health Questionnaire-9 (PHQ-9). Three-month and 6-month followup surveys of participants assess self-reported return to opioid use, non-fatal opioid overdose and engagement with SUD treatment and recovery support services. Qualitative interviews with participants, CRMs, and clinicians assess barriers and facilitators to retention in treatment and acceptability of the intervention.

#### Aim 2

Evaluate the effect of the ROAR pilot on opioid overdose among adult women released from prison. We evaluate the association between participation and the primary outcome of opioid overdose, including either a fatal or non-fatal overdose event. Fatal and non-fatal opioid overdoses are assessed for all adult females released from prison during the 18-month study period (expected *n* = 1000) regardless of participation in the ROAR intervention. Using administrative data provided by the Oregon Department of Corrections (e.g., dates of incarceration) and Oregon Health Authority (i.e., Medicaid claims and encounters), we compare the odds of overdose among participants versus a comparison group of previously incarcerated adult females with documented addiction or dependence in administrative records. Data from the comparison group will consist of de-identified administrative data for fatal and non-fatal overdoses in the state. The study will not directly recruit for this group. Statistical analyses adjust for county- and patient-level confounders including level of substance abuse treatment need and mental health treatment need assessed at prison intake.

#### Study flow

To accomplish these aims, ROAR recruits incarcerated women with diagnosed moderate to severe OUD at the end of their prison sentences. Inmates releasing to one of four pilot counties are screened for eligibility and invited to participate by prison mental health staff. Prior to release from incarceration, participants receive a single dose of XR-NTX. ROAR participants are linked with a CRM prior to release who continues to support them in community SUD treatment for up to 6 months in one of two participating treatment centers in the four pilot counties. The two partner community SUD treatment programs were selected based on their experience offering community-based re-entry services and capacity to prescribe and dispense both XR-NTX and buprenorphine. ROAR participation does not require continued treatment with XR-NTX or any other form of MOUD post release. Particpants may also choose to switch to an alternate MOUD.

**Fig. 2:**
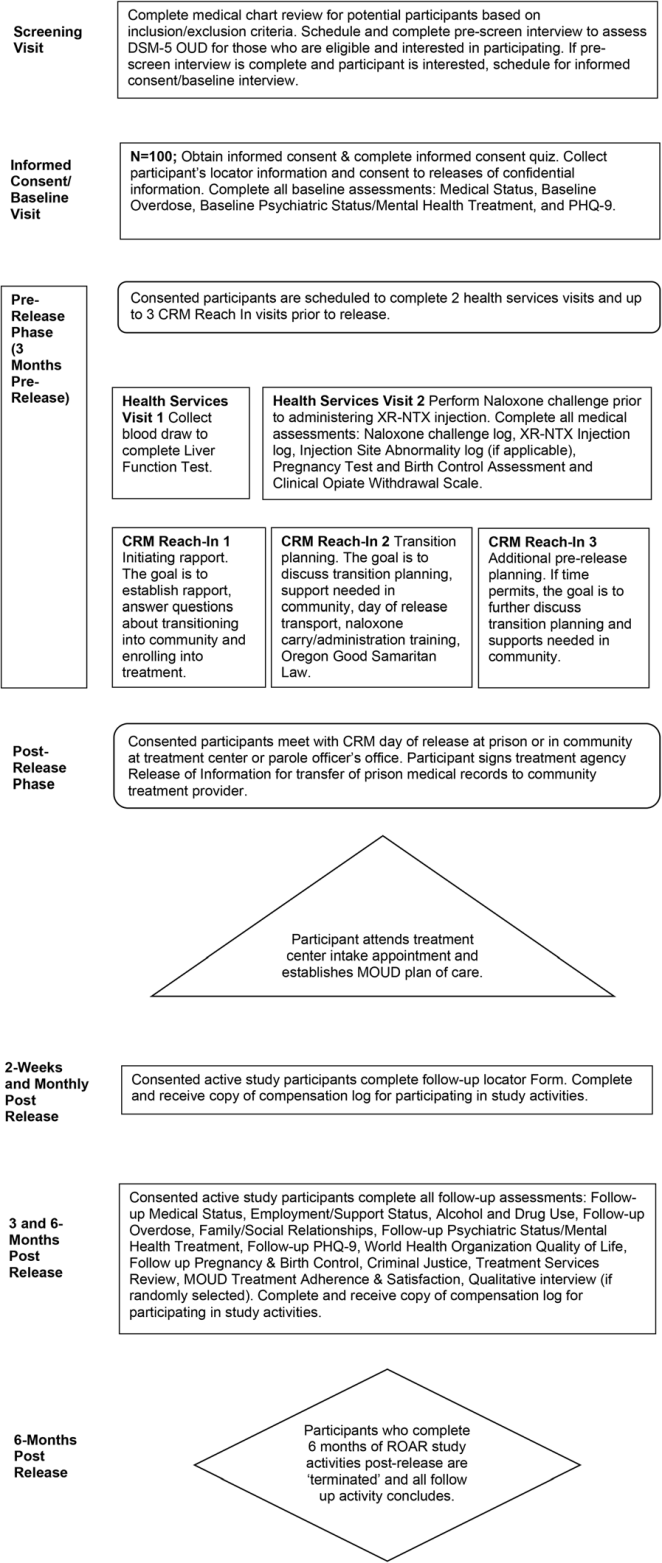
ROAR study flow

ROAR’s study flow, from screening through 6 months post release, is summarized in Fig. 2.

Study data are collected and managed using Research Electronic Data Capture (REDCap) tools hosted at Oregon Health & Science University (OHSU). REDCap (is a secure, web-based software platform designed to support data capture for research studies, providing 1) an intuitive interface for validated data capture; 2) audit trails for tracking data manipulation and export procedures; 3) automated export procedures for seamless data downloads to common statistical packages; and 4) procedures for data integration and interoperability with external sources (Harris et al., 2009; Harris et al., 2019).

### Recruitment

Development of strategies to facilitate recruitment and retention began during the study design phase. The ROAR Protocol Development Team worked closely with corrections administrators and staff representing prison operations, security and health services . Through careful deliberation and collaboration with the prison administrators and staff, we developed a recruitment process that minimizes disruption of normal facility routines and protects participant confidentiality. We work closely with key personnel at both community SUD treatment programs to ensure that the protocol accounts for the unique workforce, workflow, and other needs of each site. In order to provide autonomy for the study staff working within the prison and to reduce burden on security staff, the research team was badged to enter the prison facility and work with participants without an escort.

#### Target population

We plan to enroll 100 women in the ROAR pilot intervention during the approximately18-month recruitment period (June 2019 through December 2020). Approximately 60 adult females are released each month, half of whom are released to the four pilot counties. We expect that at least 40% will have histories of opioid abuse, providing a pool of about 12 women eligible for participation monthly, or about 3 per week. To facilitate recruitment, the Oregon Department of Corrections Office of Research, Data, & Government Efficiencies generated a list of female adults in custody who meet the following criteria: 1) have a release date during the 18-month recruitment period, 2) are being released to the four target counties, and 3) have a documented SUD. This initial list generated 2 months prior to the project launch included a total of 235 potential participants. Characteristics of the original ROAR recruitment pool are included in Table 1.

**Table 1:** Characteristics of ROAR Participant Recruitment Pool, March 2019, *N* = 235

	N (%)		N (%)
**Age Group**:		**Offense Type:**	
31 to 45	124 (52.8%)	Property	114 (48.5%)
25 to 30	54 (23.0%)	Person	69 (29.4%)
46 to 60	35 (14.9%)	Statute	52 (22.1%)
18 to 24	19 (8.1%)		
61 and Up	3 (1.3%)		
**Race/Ethnicity**:		**Drug Offense:**	
White	182 (77.4%)	None	158 (67.2%)
Black	26 (11.1%)	Some Drug Offenses	54 (23.0%)
Hispanic	16 (6.8%)	Only Drug Offenses	23 (9.8%)
American Indian	7 (1.3%)		
Asian	3 (1.3%)		
Pacific Islander	1 (0.4%)		
**Mental Health Status**:			
Severe Mental Health Problems	90 (38.3%)		
Would Benefit From Treatment	36 (15.3%)		
No Reported Need	35 (14.9%)		
Highest Treatment Need	31 (13.2%)		
Moderate Need	29 (12.3%)		
No Treatment Need	14 (6.0%)		

Additional inmates who entered the facility after the start of recruitment are continuously added to the recruitment pool. Their administrative records are pre-screened by the prison mental health counselor before being invited to screen for the project.

##### Inclusion Criteria:

- Be a woman (including transgender and non-binary adults assigned to women’s housing in prison) 18 years of age or older, with a known release date during the 18-month recruitment period
- Be released to one of four participating counties
- Be willing and able to provide informed consent and Health Insurance Portability and Accountability Act authorization for medical record abstraction and linkage to state administrative data
- Meet Diagnostic and Statistical Manual of Mental Disorders Fifth Edition (DSM-5) criteria for moderate or severe OUD
- Be willing to establish ongoing care at the community SUD treatment site in their county of release
- Be willing to initiate XR-NTX prior to release from incarceration
- Not be currently pregnant and must be willing to take at least one evidence-based measure to avoid becoming pregnant during treatment with XR-NTX
- English speaking

##### Exclusion Criteria:

- Has a severe medical, psychiatric, or SUD that, in the opinion of the prison mental health counselor or study physician, would make study participation hazardous to the participant, compromise study findings, or prevent the participant from completing the study due to imminent risk of death
- Has another medical condition leading XR-NTX to be contraindicated
- Has chronic pain requiring ongoing pain management with opioid analgesics
- Received methadone or buprenorphine maintenance therapy in the 4 weeks prior to consent
- Has a planned surgery or other procedure within the next 4 weeks that will require opioid analgesia

#### Pre-screening

The prison mental health counselor hired by the study pre-screens women with history of SUD based on intake information. Information obtained from review of administrative records is cross-referenced with a pre-screen interview which evaluates eligibility based on scheduled release date, county of release, self-reported history of opioid use in the year prior to incarceration, and diagnosis of moderate to severe OUD as assessed by the counselor.

The counselor “calls out” potential participants who pass the initial administrative record review for a one on one ROAR information session and pre-screening interview. The counselor reviews a pre-screening information sheet with participants and answers any questions. The pre-screening information sheet includes a description of the ROAR pilot intervention, what participation will involve, and privacy procedures. Those interested in participating are asked to sign the pre-screening information sheet. All participants who sign the pre-screening information sheet will proceed to the pre-screening questionnaire, which the counselor records directly in REDCap.

The pre-screening questionnaire collects age, gender identity, DSM-5 OUD diagnostic information, interest in cutting back or quitting opioid use, use of methadone or buprenorphine for the treatment of OUD, whether the participant has chronic pain that requires treatment with opioid pain medicines, whether the participant is pregnant or breastfeeding, and whether the participant would be willing to take at least one evidence-based measure to avoid becoming pregnant during XR-NTX treatment. This information is used to populate basic rule-out scenarios for those who express interest in the pilot intervention. All participants who complete the questionnaire are asked if they are interested in scheduling an appointment to participate in the ROAR pilot intervention. If they are interested, the counselor informs the participant that they will be asked to meet with study staff pre-release if they are eligible.

#### Informed consent

Those who pass the prescreening and elect to move forward are invited to meet with ROAR study staff for an informed consent and baseline interview no sooner than 90 days prior to release. ROAR participants are consented in a private area within the prison – either in the health services, education or professional visit areas. Only the participant and two study staff will be present. Screening and consent logs are maintained by study staff in REDCap and not shared with prison staff who are not engaged in ROAR.

 Study staff confirm the participant’s eligibility to participate in the pilot intervention prior to meeting and administer a short consent quiz to assess participant’s understanding of the study. If a participant provides an incorrect answer on the quiz, study staff will revisit the necessary information with the participant. Study staff will then re-ask the question, and the participant will write the correct answer on the quiz form.

Potential participants are informed that the participation in the evaluation project is voluntary and that participation will not influence the terms of their release. All inmates with OUD will receive the customary pre-release counseling, regardless of whether or not they decide to participate. At no time during the study will participants be incentivized to accept medical treatment or medication, or to meet with a CRM. Potential participants are notified that survey data will never be shared with corrections or law enforcement officials without their written permission. Participants sign the consent form to enroll in ROAR based on their willingness to receive an injection of XR-NTX and connect with a CRM prior to release to the community, and participate in research activities for up to 24 weeks post release. If a potential participant would like more time to decide to participate or to consult by phone with the study clinician, the research team and prison staff coordinate the necessary meetings to provide information to inform her decision.

### ROAR intervention

The pre-release phase includes screening and medication initiation activities, which are briefly described below. The post-release phase includes survey and interview data collection and regular participant check-ins, along with community treatment and ongoing support from CRMs. Return to opioid use is an expected part of the recovery process, and all participants are released with nasal naloxone rescue kits. The naloxone kits are reviewed with the participants during the health services visit, at day of release with the CRM, and throughout their follow up in community to assess the need for ongoing education, training, and whether or not the naloxone has been used and there is need for an additional rescue kit. Nasal naloxone is a medication that cannot be abused and has no “street value” in terms of the being sold or traded in community, therefore, there is limited risk of it being sold or stolen. Participants who return to opioid use remain eligible to continue participation in ROAR, to receive support from their CRM, and are contacted for participation in the 3-month and 6-month follow-up interviews. All study procedures and assessments are summarized in Table 2. Note that variation in the timeline is expected, based on changes in participant release dates. If a participant receives an injection of XR-NTX more than 3 weeks prior to release (due to unexpected change in release date), then the participant will be maintained on XR-NTX with monthly injections up to the week prior to actual release.

**Table 2:** ROAR Assessments and Study Activities

	Pre-Release ROAR Activities								Release Day	Post-Release ROAR Evaluation Visits						
Tests/ Procedures	Prescreen		Consent & Baseline	CRM Reach In Visits			Health Services			2 Week Check In	1 Month Check In	2 Month Check In	3 Month Follow Up (& Qualitative Interview)	4 Month Check In	5 Month Check In	6 Month Follow Up
**Study Visit Number**	**PS1**	**PS2**	**Consent & Baseline Interview**	**CRM1**	**CRM 2**	**CRM 3** **(optional)**	**HS1**	**HS2**	**V0**	**V1**	**V2**	**V3**	**V4**	**V5**	**V6**	**V7**
**Approximate Weeks Post-Release**	**−12**	**−8**	**−6**	**−4**	**−2**	**−1**	**−4**	**−1**	**0**	**2**	**4**	**8**	**12**	**16**	**20**	**24**
Medical Chart Review	**X**															
Prescreen Interview (DSM-5 OUD)		**X**														
Inclusion/Exclusion Checklist		**X**														
Demographics (Informed Consent and Consent Quiz)			**X**													
Locator Form			**X**													
Medical Status			**X**													
Baseline Overdose			**X**													
Baseline Psychiatric Status/Mental Health Treatment			**X**													
PHQ-9			**X**													
Liver Function Test							**X**									
Pregnancy Test and Birth Control Assessment								**X**								
Naloxone Challenge Log								**X**								
Clinical Opiate Withdrawal Scale								**X**								
XR-NTX Injection Log								**X**								
Injection Site Abnormality Log								**X**								
Follow Up Locator Form										**X**	**X**	**X**	**X**		**X**	**X**
Follow-up Medical Status													**X**			**X**
Employment / Support Status													**X**			**X**
Alcohol and Drug Use													**X**			**X**
Follow-up Overdose													**X**			**X**
Family/Social Relationships													**X**			**X**
Follow-up Psychiatric Status/Mental Health Treatment													**X**			**X**
Follow-up PHQ-9													**X**			**X**
World Health Organization Quality of Life-8													**X**			**X**
Pregnancy & Birth Control													**X**			**X**
Criminal Justice													**X**			**X**
Treatment Services Review													**X**			**X**
MOUD Treatment Adherence & Satisfaction													**X**			**X**
Qualitative interview (if randomly chosen)													**X**			
CRM Reach-In Log				**X**	**X**	**X**	**X**	**X**	**X**	**X**	**X**	**X**	**X**	**X**	**X**	**X**

#### Staff training

All staff who interact with participants or participant data are certified by CITI in human subjects’ protection, good clinical research, responsible conduct of research, and OHSU conflict of interest. All research staff collecting data on site in the prison complete in-person and on-line training modules required of corrections volunteers and consultants, including a thorough facility orientation at the prison. In the month before the project launch, all Department of Corrections, treatment center and research partners convened for a full day in-person ROAR orientation at the prison. Topics included the study rationale, intervention and data collection procedures. Study clinicians presented an overview of MOUD and its administration in correctional settings. Nursing staff received additional in-person clinical training from Alkermes, the manufacturer of XR-NTX . Study clinicians from OHSU and the NYU Grossman School of Medicine provide ongoing clinical support as needed. Comagine Health provides weekly phone meetings and at least quarterly in-person training on topics essential to provision of peer support within populations of justice-involved women (e.g., Gender Specific & Trauma Informed Services, Criminality and Prosocial Behaviors, Social Learning Theory, Prison Rape Elimination Act (PREA), Co-Occurring Disorders in OUD and Naloxone & Overdose Prevention).

##### CRMs

ROAR’s two partner community treatment programs employ grant-funded female ROAR mentors who are CRMs in the State of Oregon. They meet with ROAR participants during the release planning process and in the community during the 6 months post release while enrolled in the pilot intervention. CRM visits in community are partially reimbursed by state Medicaid, once the participant has enrolled in treatment services at the treatment agency.

A CRM must complete a State of Oregon Behavioral Health Division-approved training program. CRMs are a self-identified person in recovery from a SUD and must meet the abstinence requirements for recovering staff in Oregon’s alcohol and other drug treatment programs (i.e., attestation that CRM has not used alcohol or illicit drugs or abused prescription medication in the two years immediately preceding employment). CRMs in Oregon complete a minimum 40 h of training and undergo a criminal background check and a national psychometric examination in order to obtain state certification. ROAR CRMs have additional training provided by Comagine Health in criminality theory and the role of the forensic peer, gender-specific needs and service delivery, including co-occurring disorders, trauma-informed services, physical health considerations for women, social learning theory, criminality and pro-social behaviors, PREA, and relapse and overdose prevention. CRMs are trained to administer and carry nasal naloxone rescue kits. CRMs are evaluated on the Substance Abuse and Mental Health Services Administration (SAMHSA’s) twelve core competencies for peer workers in behavioral health services throughout the study (SAMHSA, 2015).

Clinical supervision for the ROAR CRMs is provided by the SUD treatment program supervisors. CRMs utilize the SUD Peer Supervision Competencies and the Forensic Peer Best Practices Curriculum as the foundation for agency supervision and monitoring. Fidelity monitoring and supervision to the CRMs is by Comagine Health. ROAR CRMs located in all four counties have weekly Skype meetings facilitated by the Research Team at Comagine Health. These meetings review study protocols, peer mentoring topic areas related to the study participants, discuss barriers or potential pitfalls to the model, and provide interdisciplinary support. CRMs attend in-person MetroPlus Association of Addiction Peer Professionals meetings once a month in the Portland Metro area. This is a monthly meeting of recovery mentors in the tri-county area that provides ongoing CEU trainings, resource sharing, and up to date best practices for SUDs and the role of the peer mentor. Only female mentors are employed in the pilot in order to gender match which has been found to increase alliance and retainment in services. Additionally, mentors only have a ROAR caseload of up to 25 participants in order to be available for the required pre and post incarceration activities.

#### Pre-release procedures

##### Health services visit 1

Approximately 1 month prior to release, consented participants attend an education and counseling appointment with a corrections health services nurse. During this visit, the nurse f reviews the risks and benefits of treatment with XR-NTX and asks the participant to share her understanding of the study medication to ensure that she fully understands how XR-NTX may affect her. In addition, the nurse explains the required naloxone challenge and pregnancy test to the participant. The nurse also draws blood for a liver function test. Lab tests are collected and reviewed by corrections health services, prior to prescription of XR-NTX by the Department of Corrections Medical Director. The ROAR study clinician housed at OHSU provides medical consultation to corrections health services staff as needed to confirm the participant's eligibility to receive XR-NTX.

##### Health services visit 2

The nurse schedules the injection visit with corrections health services 3 to 7 days prior to the participant’s scheduled release. Prior to the injection of XR-NTX, the nurse administers a nasal naloxone challenge and a pregnancy test.

In lieu of urinalysis, which could place participants at risk for sanction if positive, a naloxone challenge (using nasal spray) is used to confirm that an injection of XR-NTX would not precipitate opioid withdrawal. As intra-nasal naloxone rescue kits are provided to all ROAR participants at release, the participant is instructed in the administration of the nasal spray during this time. The nurse administers the first spray, and the participant self-administers the second spray. After the naloxone is administered, participants are monitored for opioid withdrawal symptoms for up to 1 hour. If opioid withdrawal symptoms are present, the participant will be treated by health services, and no XR-NTX injection will be given. In the case of a failed naloxone challenge, the participant will be advised that they can remain in the study, and the study CRM will facilitate linkage to appropriate treatment in the community.

 XR-NTX is not indicated for pregnant or nursing women, so a pregnancy test is administered in corrections health services prior to injection. A new positive pregnancy test prior to release would be a serious adverse event. The ROAR nursing staff would communicate the result to the Health Services Manager, who woulddetermine appropriate procedures, in line with Department of Corrections policies and in compliance with PREA.

 Immediately prior to the injection, participants again review the risks and benefits of the medication with nursing staff, including a potential increased risk of overdose if a dose is missed or after stopping treatment. If no opioid withdrawal symptoms are present, a single 380 mg XR-NTX dose is administered intramuscularly to the right upper outer gluteus by a trained corrections health services nurse. Additional training on XR-NTX and nasal naloxone is provided to the ROAR study team by the study clinicians. The injection site and any side effects are assessed during the final week of incarceration as needed.

Consented ROAR participants who do not receive an XR-NTX injection for any reason may choose to continue in the ROAR evaluation, and remain eligible for services provided by the CRM and facilitated entry into community SUD treatment.

##### CRM reach-in visits

Consented ROAR participants are introduced to CRMs approximately 1 month prior to release. CRMs meet with the participant up to three times prior to release. Meetings take place in person in visiting areas of the prison. These initial meetings provide an opportunity for the mentor to establish rapport with the participant, clarify further questions about the study, discuss expectations of pre-release ROAR activities, address concerns about re-entry into the community, and discuss plans to link the participant to ongoing SUD treatment services, including MOUD. The goal of the initial meeting is to establish rapport, answer questions about transitioning into community and enrolling into treatment. Subsequent pre-release CRM visits may focus on transition planning. The CRM and participant meet to discuss transition planning, support needed in community, and day of release transport. Naloxone carry/administration training includes an overview of the Oregon Good Samaritan overdose law, which protectst against arrest or proscution for drug possession or being in a place where drugs are used when medical help is sought for overdose.

#### Post-release procedures

##### SUD treatment in community

Participants are encouraged to engage in SUD treatment within the first 4 days of release and are followed by research staff and CRMs for 24 weeks. Participants may receive follow-up medical care, including any subsequent XR-NTX, buprenorphine or methadone, in community. Follow-up medical appointments within 1 week of release and any prior authorization for continued medication are part of release planning for ROAR participants. Subsequent XR-NTX injections are offered at the community treatment centers, and covered by Medicaid. The CRM engages with the participant prior to release and upon release to facilitate follow-up care. Participants are free to choose whether to continue XR-NTX or switch to opioid agonist therapy with buprenorphine or methadone following their release. Both participating treatment centers offer XR-NTX and buprenorphine on site, as well as either referral or delivery of methadone maintenance. The participant may also choose to discontinue medication treatment, while participating in outpatient or other counseling services.

##### CRM support in community

The CRM works directly with the prison counselor to coordinate post-release activities. Participants have direct contact with the CRM , at day of release. The CRM provides transportation to the participant’s parole officer and intake session at the community treatment provider, unless alternative transportation arrangements are made with the participant’s family or another program. The CRM coordinates the participant’s initial assessment at the treatment center and may accompany the participant to their first mutual aid support meeting (e.g., 12 Step, Smart Recovery, Women for Recovery, Refuge Recovery) if desired.

The CRM assists the released participant in establishing recovery goals and supports a wide range of activities, including linkage to resources (e.g., obtaining identification card, food stamps, supportive employment, parenting support groups) and accompanying the participant to their first recovery support meeting if desired. The CRM aids the participant with ongoing assessment of safe and stable housing along with the continued goals of supporting pro-social behaviors and addressing criminogenic risk factors such as criminal thinking and antisocial attitudes and beliefs. CRMs and study participants have frequent contact (recovery check-ins) based on the needs of the participant (daily, weekly, or bi-weekly) during the study follow up period. Recovery mentor supports are individualized, however, contact with the CRM is expected to be frequent during the first 2 weeks post release and as agreed upon by the participant and the CRM.

CRMs provide hope, empowerment, support, advocacy, and education utilizing person-first language through unconditional positive regard, self-disclosure and honoring that there are many paths to and stages of recovery. Frequency of mentor services provided during the pilot are dependent on the American Society of Addiction Medicine level of care at which the participant is assessed, including areas of need outlined in the participant’s treatment plan. CRM services (frequency and duration) are documented in the participant’s treatment plan and reviewed for continuation of care by the treatment center.

##### Participant retention and compensation

Retention is a vital part of any intervention protocol and being able to contact a participant is instrumental to high retention rates. Thus, it is critical to have each participant complete a locator form and update the form as needed throughout the pilot intervention and evaluation. The locator form is initially completed immediately following informed consent, and participants are queried for any changes during each follow up activity. When completing the locator form, participants provide their names and any aliases, current address, email address, and telephone numbers for themselves and (if possible) at least two other people. C At time of consent, participants are provided a ROAR contact card that includes the name, email address and cell phone number for research staff that will complete the follow-up check-ins and interviews. Participants are also reminded that CRMs can share their contact information with research staff. This is important because CRMs complete reach-in appointments close to date of release when participants may have additional contact information and often meet participants on their day of release. Research staff and CRMs communicate frequently about updated participant contact information.

Barriers to retention in the ROAR pilot are similar to barriers to retention in SUD treatment programs and include return to opioid use, transportation costs, competing resources, mistrust of medical providers and research staff, fear of stigma, and concerns about the medication and potential side effects. Frequent contact with the CRM likely increases study retention. Connections to the CRM provide participants with opportunities to clarify processes, and reduce uncertainty or fears about participation in the pilot program.

Participants are not compensated for meetings with their CRM or any medical or behavioral health treatment in the community, or for any study activities conducted while incarcerated. Participants are paid up to 150 USD over 6 months after release from incarceration for participating in check-in calls and completing brief surveys and qualitative interviews. Payments are in the form of gift cards. Participants receive 10 USD gift cards to check in with the Research Coordinator and update their locator forms at weeks 2, 4, 8, 16 and 20. For the check-in, participants meet with research staff in person, by phone, text message or email to confirm their contact information and schedule appointments. Participants receive a 25 USD gift card for completion of the 3-month follow-up assessments and a 50 USD gift card for completion of the 6-month follow-up assessments. Participants are not required to attend any clinical visits in the community or to meet with their CRM in order to participate in the follow-up assessments. 

Participants who experience adverse reactions or poor response from the clinical staff may be more likely to drop out of the pilot program. The study team has developed procedures to reduce the risk of adverse events and ensure appropriate and timely management and follow-up when adverse events occur. It is expected that some participants may wish to discontinue study participation because they may not want to continue XR-NTX. Participants are assured that they are free to decline injections of XR-NTX and explore other treatments or no treatment while remaining in the study. Finally, recruitment and retention are regular topic at weekly team meetings that include key personnel at the prison and the two community SUD treatment programs, as well as ROAR study staff.

### Preliminary enrollment and retention data

**Fig. 3:**
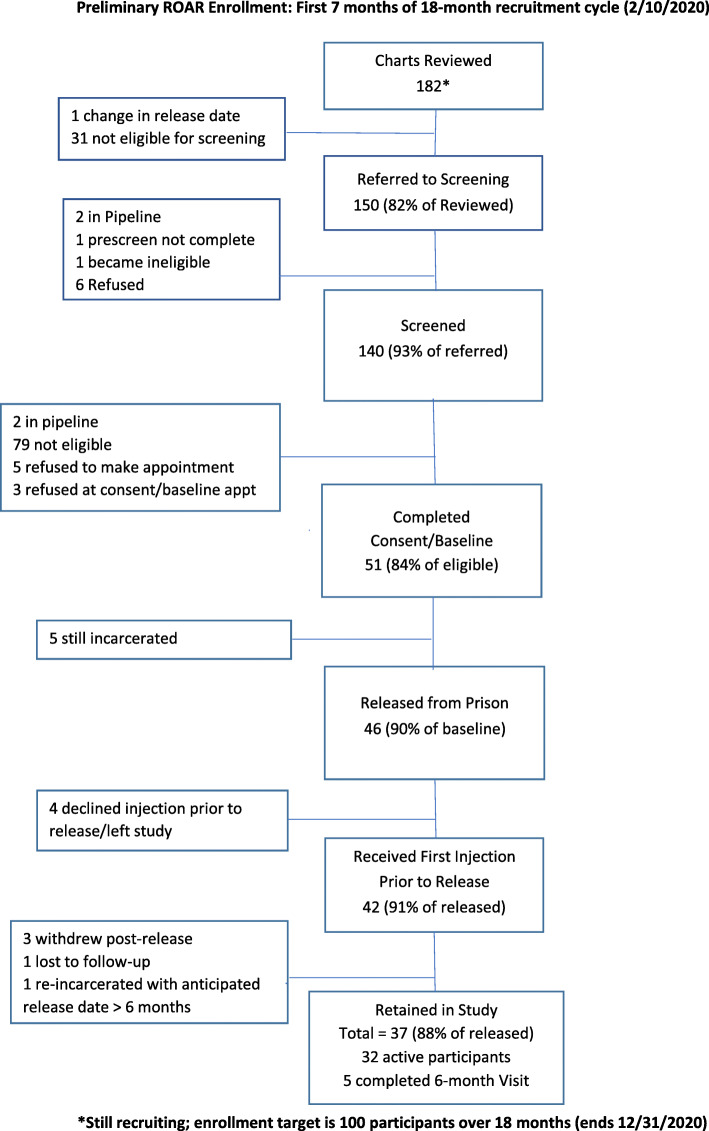
Preliminary ROAR enrollment: first 7 months of 18-month recruitment cycle (2/10/2020)

Enrollment and retention data for the first 7 months of recruitment are summarized in Fig. 3. At the time of publication, new enrollments were paused due to the Coronavirus (COVID-19) pandemic. Oregon Department of Corrections closed its facilites to non-essential staff and suspended all  visiting on March 13, 2020. Enrolled ROAR participants continued to receive follow up medical treatment and remote CRM services via secure video conference, phone, email, and text. Research check-ins, 3-month and 6-month follow-up surveys, and qualitative interviews continued by phone or secure videoconference.

Early recruitment and retention rates suggest the that ROAR pilot will successfully enroll and retain participants as planned. Of 150 adults in custody referred for prescreening interview by end of month 7, 93% were screened using an in-person interview guide. Table 3 summarizes reasons why 32 women in custody were excluded from pre-screening interview process. Of those who completed the pre-screen interview (n=140), 61 were eligible to enroll. At total of 51participants (84% of those eligible at pre-screen) were consented and enrolled into the pilot. Of the first 46 participants released from prison, 42 accepted the first injection of XR-NTX prior to release. Of those, 86% were retained as participants in community (31 still active, and 5 completed 6-month follow-up). Recruitment is planned for a total of 18 months.

**Table 3:** Ineligibility for ROAR Pilot Project (First 7 Months Recruitment)

	Number
**Reasons ineligible for pre-screening, post administrative record review (multiple response)**	32
Detainers	14
Mental Health	10
Medical Concerns	6
Refused prior to being presented with information sheet	5
Special Housing	2
Change in Release Date	1
**Reasons Ineligible for Consent/Baseline, post Screening Interview (multiple response)**	79
Does not meet DSM-5 criteria for moderate to severe opioid use disorder	68
Not interested in obtaining treatment to cut back or quit opioid use or to prevent using opioids when released	68
Would not consider medication-based treatment for opioid use disorder	36
Not willing to take at least one evidence-based measure to avoid becoming pregnant	11
Chronic pain that requires ongoing treatment with opioid pain medicine	7
Release outside of target counties	5
Disqualified by prison health services	2
Planning on conceiving in the coming months	1

### Data collection and measures

Our primary outcome is any opioid overdose (fatal or non-fatal) within 6 months following release from incarceration. This is a binary outcome indicating a fatal or non-fatal opioid-related overdose event in the 6 months following release from incarceration. We identify fatal opioid overdose with linked vital statistics data and define fatal overdose using International Classification of Disease, 10th Revision specific codes for opioid poisoning. Non-fatal overdose is measured using Oregon Medicaid and hospitalization discharge data. We use the date of admission as the overdose date. Emergency department encounters occurring within 1 day of a hospitalization are not counted as distinct overdose events. As secondary analyses, we will examine which baseline characteristics are associated with implementation and client outcomes using both descriptive statistics as well as bivariate methods.

#### Baseline Surveys and Assessments

During the informed consent/baseline data collection visit with the ROAR staff, participants are asked to complete baseline surveys, which provide individual characteristics that will be used to describe study participants and explore factors potentially associated with implementation and client outcomes. The Baseline Survey collects demographic information, medical status, overdose experiences, psychiatric status and mental health treatment, and PHQ-9 responses. Selected items from the Addiction Severity Index Lite (ASI Lite) will assess an individual’s history related to substance use, family/social status, employment, and medical and psychosocial issues.

*Questionnaires Included in Baseline Survey:*

- PHQ-9
- Participant Demographics
- ASI Lite (selected items)

#### Follow-up Surveys

At 3- and 6-months post-release, participants are invited to complete a 30-minute face to face survey that collects information about medical status, employment/support status, alcohol and drug use, overdose experiences, family/social relationships, psychiatric status and mental health treatment, patient health questionnaire responses, quality of life, criminal justice involvement, treatment services review, and MOUD treatment adherence. The ROAR research coordinator records participant responses directly in REDCap. The survey meetings are conducted in person in a public place of the participant’s choosing.

Items include the ASI-Lite questions adapted for a 3-month timeframe and the overdose experience items. Quality of life is measured using the World Health Organization Quality of Life-8 . Items using previously tested language will capture overdose experience. Participants are compensated 25 USD for completing the 3-month survey and 50 USD for completing the 6-month survey. Information collected from surveys is not shared with law enforcement officials.

##### Questionnaires Included in Three and 6 Month Follow-Up Surveys:

- ASI Lite
- Criminal Justice
- WHOQOL-8
- Treatment Services Review
- MOUD Treatment Adherence and Satisfaction (MOUD)
- Pregnancy and Birth Control
- PHQ-9

##### Qualitative Interviews

A random sample of consented participants are invited to complete a qualitative interview 3 months post release. We use the block randomization (block size of 3 from each sequential group of 10 consented participants). Semi-structured interviews with participants explore acceptability, patient-centeredness and satisfaction, including perceived utility of recovery mentor services, and barriers and facilitators to retention treatment. CRM and clinician interviews examine the implementation factors such as organizational setting, characteristics and implementation outcomes. All interviews are audio recorded, professionally transcribed and uploaded into a secure server for analysis. Interviews will last 60 minutes or less, and ROAR participants are compensated an additional 25 USD.

ROAR CRMs and clinicians are asked to complete qualitative interviews during the 18-month study period during work hours and are not compensated. Interviews will take place during the second half of program implementation.

##### Service Utilization Data

 Clinic staff abstract service utilization records from ROAR clinic sites documenting receipt of XR-NTX or other MOUD. Evaluation variables will include self-refort of engagement and retention in peer and SUD treatment services. CRMs log every interaction with participants, including reach-in visits, release visits, community meetings, phone calls, text messages and emails. The duration and purpose of each encounter is logged in REDCap using a structured tool.

##### Limited Administrative Dataset

Fatal and non-fatal opioid overdoses will be assessed for all women released from prison during the 18 month study period (expected *n* = 1000) regardless of participation in ROAR. Using administrative data provided by the Oregon Department of Corrections and the Oregon Health Authority, study staff will compare odds of overdose among ROAR participants (expected n = 100) versus a comparison group of at least 100 previously incarcerated adult women with a documented SUD who are released into Oregon counties that are not participating in ROAR. Multivariate statistical analyses will be conducted to adjust for county and patient level cofounders including level of substance abuse and mental health treatment needs assessed at intake.

#### Analysis

##### Opioid Overdose

The primary outcome, opioid-related overdose, is measured using administrative (Medicaid claims, hospital discharge data) and vital statistics. We use hospital discharge data to supplement Medicaid claims data in the event that participants lose or change Medicaid enrollment. The validity of administrative data to identify opioid-related overdose is high (sensitivity = 97%, specificity = 85%) (Green et al., 2019). We use Oregon vital statistics (death certificate data) to ascertain opioid-related fatalities. Oregon has a centralized medical examiner model which is known to provide more consistent and specific reporting of drugs implicated in fatalities (Buchanich, Balmert, Williams, & Burke, 2018; Warner, Paulozzi, Nolte, Davis, & Nelson, 2013).

Our primary analysis will examine the effect of pilot participation on the rate of fatal and non-fatal overdose events. We will calculate person-years of follow-up using the time between release date and subsequent death or study conclusion. Thus, women will contribute to calculations of person-years once they are released, except during subsequent incarcerations or after fatality. We will censor follow-up at the end of Quarter 10 or the date of incarceration if they were in prison as of this date. Our primary outcome (any opioid overdose) will be modeled as a rate with person-years as an offset variable.

We will employ either a mixed logistic model if the outcome turns out a non-rare event, or a mixed poisson model (with poisson approximation to binomial distribution) if the outcome turns out to be a rare event. The effect of ROAR participation will be examined by testing the slope coefficient (i.e., b1, see below) of the main treatment effect variable (ROAR participation yes/no) in the model. We will also adjust the treatment effect using covariates such as age, race/ethnicity, incarceration time, and mental health level of treatment need while incarcerated. We control for correlation due to clustering at county level by including a random effect (i.e., α, see below) in the model.

*Alternatively, the model described below is employed for our analysis:*

g[E(y|x)] = b0 + b1*ROAR,+b2*x + α, where g(.) is a link function (e.g., logit or log link), and b2 is a slope coefficient vector of adjusting covariates included in the model; α is the random effect account for county specificity.

As an exploratory analysis, we will investigate which baseline characteristics are associated with any overdose as our primary outcome measure, in a multivariable regression. Variables will be considered for inclusion as covariates in a final multivariable models if they are found to be associated with any overdose event in bivariate analyses at a *p* < 0.25 threshold.

##### Implementation

We evaluate the implementation of ROAR (acceptability, adoption, retention, and engagement), service outcomes (service intensity) and client outcomes (overdose, return to substance use, and quality of life). All outcomes will be presented as a percentage of the women who consent to participate in ROAR, except for acceptability (in which the denominator will be eligible inmates with documented moderate to severe OUD at pre-screening), and quality of life (which is a continuous measure). Outcomes measured at months three and six (retention, self-reported overdose, return to substance use, and quality of life) will be compared over time to determine if the likelihood of these events changes as participants are in the community for a longer period. We will compare these longitudinal outcomes measured as proportions using a McNemar’s test. Since quality of life at baseline as well as months three and six will be measured, repeated measures Analysis of Variance will be used to assess change over time.

We will assess the association of baseline characteristics with implementation and client outcomes using both descriptive statistics as well as bivariate methods. Bivariate methods will be selected as appropriate for each variable; they may include but are not limited to chi-squared tests, fisher’s exact, t-test, Mann-Whitney U, Pearson’s and Spearman’s correlation coefficients, etc.

Qualitative data will be entered into NVivo qualitative software and coded by two research staff using an iterative codebook created by the research team. . This method will be conducted via group process, with reliabilty checks conducted by the lead qualitative researcher (Baker).. These processes will continue until patterns and inferences are well articulated. Qualitative findings will be triangulated with quantitative data collected for Aim 2 to support and explain intervention effectiveness, by providing evidence related to intervention acceptability and feasibility; implementation quality, intensity, and barriers; and contextual influences.

##### Sample Size and Power

Based on current release data, we expect to enroll approximately 100 participants over the 18-month recruitment period. At least 100 non-participating formerly incarcerated women with SUD released from prison over the same time period comprise our comparison group, which is built from Oregon state administrative data. Using Markov chain Monte Carlo simulation, the minimal detectable difference between ROAR and non-ROAR groups with 80% power, 5% type I error, and equivalent sample of *n* = 100 in each of the ROAR and non-ROAR groups was computed. It was assumed that varying baseline rates of post-release overdose ranging from 20% and 25% per year, based on data from Oregon Health Authority and the increased risk of overdose in similar populations as reported in the literature. Note that this power analysis has taken into consideration the correlation due to clustering within county level. There are a few scenarios of common correlation for clustering in practice that have been considered while performing the power analysis. Raw risk difference is defined as a raw difference (e.g., p1-p2), and relative risk difference is defined as (p1-p2)/p1.

In the Table 4, we summarize minimum detectable absolute differences with associated relative risks, accounting for varying assumptions about within-county clustering (i.e., correlation).

**Table 4:** ROAR power analysis

Minimal detectable differences of post-release opioid overdose as absolute difference and relative risk				
	Baseline rate 20%		Baseline rate 25%	
ρ	Raw risk difference	Relative risk difference	Raw risk difference	Relative risk difference
0.0000	0.105	0.48	0.12	0.52
0.0050	0.115	0.43	0.125	0.50
0.0075	0.120	0.40	0.13	0.48
0.0100	0.120	0.40	0.135	0.46

Calculations assume equivalent sample size of 100 participants for each control and intervention groups, a minimum of 80% power, and alpha level of 5%

Programs that facilitate MOUD in correctional facilities have been previously shown to have a large effect on overdose mortality (RR = 0.4). Given the assumptions described, our pilot is sufficiently powered to detect treatment effects similar to what has been observed in other studies of MOUD delivery through the correctional system (Green et al., 2018).

## Discussion

Oregon is well-positioned to successfully pilot and maintain a new model of pre-release linkage to MOUD and CRM services for incarcerated individuals. The state has a lengthy history of demonstrated support for peer mentor services and a network of more than 1,200 CRMs. Oregon Medicaid covers peer support services and has an established peer training and certification program overseen by Oregon’s addiction professional licensing board and an active peer professional association. In 2017, a statewide expert workgroup developed a peer supervision competency guide and a best practice guide for forensic peer specialists working with individuals with criminal justice involvement. Similar to many other states, however, standardized pre-release peer linkages have not been established for incarcerated individuals. In 2017, Oregon’s Governor Kate Brown established a statewide opioid taskforce with a focus on increased access to opioid agonist and antagonist therapy and overdose prevention. The taskforce sponsored a successful bill providing funds to study and address barriers to MOUD and strengthen peer recovery support programs. The legislation requires the Oregon Health Authority, Department of Corrections, and Department of Consumer & Business Services to collaborate to identify these barriers to MOUD.

 ROAR's pragmatic evaluation plan has a number of inherent limitations. Our analysis of opioid-related overdoses using administrative data is an observational cohort design. Although we use a concurrent comparison cohort of women with OUD released into non-ROAR counties as a comparison group, these women may systematically differ in ways related to our outcomes. We attempt to mitigate this limitation by using multivariable regression models to control for potential imbalance, but residual confounding may persist. We will be circumspect when reporting findings from this analysis and acknowledge this limitation.

The current study’s findings with regard to induction, adherence and effectiveness of XR-NTX among justice-involved women may not be generalizable beyond prospective cohorts of incarcerated women who are started on XR-NTX before release. There is an underlying assumption that participants will not require detoxification prior to starting XR-NTX in a prison population. Retention on medication in community is dependent on available treatment providers and coverage of the cost of medications. In the current study, partnering agencies already offered treatment with MOUD that is covered by local Medicaid. Enrollment and transportation to treatment agencies is facilitated by CRMs, whose services are also reimbursable by local Medicaid. The pilot program excludes pregnant women and those planning to become pregnant, as XR-NTX is not indicated in pregnancy. This is a major limitation of the project, as it does not address the treatment needs of pregnant women or those planning pregnancy soon after release from prison.

The state of Oregon has recognized the need to strengthen cross-disciplinary partnerships and its Department of Corrections is prepared to begin playing a role in preventing overdose for a particularly vulnerable population: women with OUD preparing for community re-entry. Successful implementation of ROAR can set the stage for expanding the initiative to include men released from prison. ROAR’s evaluation leverages Oregon’s capacity for complex administrative data linkages to pragmatically assess strategies to reduce risk of opioid overdose among previously incarcerated women.

## Data Availability

Not applicable.

## Abbreviations

PHQ-9: Patient Health Questionnaire-9
ROAR: Reducing Overdose After Release from Incarceration
REDCap: Research Electronic Data Capture
ASI-Lite: Addiction Severity Index Lite
CRM: certified recovery mentor
DSM-5: Diagnostic and Statistical Manual of Mental Disorders Fifth Edition
MOUD: medications for opioid use disorder
OHSU: Oregon Health & Science University
OUD: opioid use disorder
PREA: Prison Rape Elimination Act of 2003
SAMHSA: Substance Abuse and Mental Health Services Administration
SUD: substance use disorder
XR-NTX: extended release naltrexone
